# Active touch sensing: finger tips, whiskers, and antennae

**DOI:** 10.3389/fnbeh.2014.00050

**Published:** 2014-02-20

**Authors:** Robyn A. Grant, Pavel M. Itskov, R. Blythe Towal, Tony J. Prescott

**Affiliations:** ^1^Division of Biology and Conservation Ecology, Manchester Metropolitan UniversityManchester, UK; ^2^Champalimaud FoundationLisbon, Portugal; ^3^QualcommLa Jolla, San Diego, CA, USA; ^4^Department of Psychology, University of SheffieldSheffield, UK

**Keywords:** sensorimotor, animal models, sensation, active sensing, touch

Active touch can be described as the control of the position and movement of tactile sensing systems by reaching out and exploring—sensing by “touching” as opposed to being touched. The active nature of these movements entails precise control of the sensory apparatus, which is task-specific and maximizes sensory information from the environment. This collection brings together a group of articles from progressive, early career scientists who are researching active touch sensing from a variety of different perspectives including behavioral, physiological, neuronal, computational, and robotic. There are a host of different model systems that are used to investigate active touch sensing and this collection sees the three main systems represented; that of the human hand, mammalian whiskers, and insect antennae. In this collection we have grouped the studies together into sections by their system and each section contains a collection of articles from both Frontiers in Behavioral Neuroscience and Frontiers in Neurorobotics. We feel that this indicates the truly multidisciplinary nature of studying active touch.

The first section of articles covers various aspects of human touch. As with all active touch systems, humans move their fingertips through a sequence of exploratory movements that yields the most information from the task. These movements depend on both the task in hand and prior experience. Ackerley et al. (2012), demonstrated using fMRI that different brain areas are functional in active touch tasks compared to passive touch ones. Articles within this section of the collection demonstrate that biomimetic robots can exploit the information generated by different movements to select the most “useful” movements that maximize information to successfully discriminate between different textures (Fishel and Loeb, 2012; Pape et al., 2012) and are able to judge surface compliance (Su et al., 2012). Texture tasks can be achieved by processing sensor information from fingertips, but also the movements of the wrists and fingertips in relation to the surface, which can code for surface properties (Delhaye et al., 2012). Klöcker et al. (2012) show that as well as movement, force and vibration data, touch can also be related to feelings of pleasantness, with fingertip moisture levels as a good correlate of unpleasantness.

The whisker, or vibrissal, system of small mammals is an important model of active touch sensing. The majority of the work in this area is aimed at understanding the neural substrates that are involved in this complex sensorimotor system. In order to obtain reliable tactile information about their environment most of the whiskered mammals move their vibrissae rhythmically, a motion known as whisking. Unlike in human touch, the whiskers are ultimately made up of dead cells, with sensory information obtained at the base of the whisker, in the follicle. Boubenec et al. (2012) propose that the contact and detachment of a whisker with a surface is likely to give rise to the most significant neural responses. Changes in the bending moment of the whisker can then be used to calculate the position of contact with an object along the whisker, and used to predict the object curvature and translation (Schroeder and Hartmann, 2012). However, predicting the position of object contact from bending moments is complex, and changes with whisker velocity (Evans et al., 2013). As with humans, movements of the sensory apparatus, the whiskers, are dependent on prior experience. Small mammals have functional whiskers from birth. Grant et al. (2012) show that rats orient to whisker contacts from conspecifics from a young age to maintain aggregations, indicating the crucial role the whiskers play in development. Anjum and Brecht (2012) show that young shrews can use their whiskers to locate and hunt crickets in an adult manner, and can moderate their attack strategy to novel prey items. This demonstrates that hunting behavior is both innate, subject to modification from experience and reliant on active touch.

In our final section we look for inspiration in the insect antennal system. Insects have a pair of antennae on their head that are involved in a range of sensory-guided behaviors. Just like with the mammalian whiskers, these antennae can be used to locate the position of an object contact along the antennae, discriminate between surfaces and their sensory information is also affected by velocity (Pape et al., 2012). However, as well as object discrimination and orienting to prey and conspecifics, tactile information can also be used to guide locomotion. Krause and Dürr (2012) show that antennal touches guide the positions and speeds of joint movements as stick insects step over objects.

We believe that this collection of papers on humans, small mammals, and insects represents the current state of research in active touch sensing. As biologists, neuroscientists, roboticists, and computational modelers continue to work together, we predict that this field will continue to expand and hope to see more active touch behaviors defined and characterized in the future; with an emphasis on their function in the behaving animals. We hope that you enjoy reading this collection of papers as much as we have enjoyed putting them together.
